# Intelligent Coaching Assistant for the Promotion of Healthy Habits in a Multidomain mHealth-Based Intervention for Brain Health

**DOI:** 10.3390/ijerph182010774

**Published:** 2021-10-14

**Authors:** Diego Moreno-Blanco, Javier Solana-Sánchez, Patricia Sánchez-González, Manuel Jiménez-Hernando, Gabriele Cattaneo, Alba Roca, Joyce Gomes-Osman, Josep María Tormos-Muñoz, David Bartrés-Faz, Álvaro Pascual-Leone, Enrique J. Gómez

**Affiliations:** 1 Biomedical Engineering and Telemedicine Centre, ETSI Telecomunicación, Center for Biomedical Technology, Universidad Politécnica de Madrid, 2804 Madrid, Spain; p.sanchez@upm.es (P.S.-G.); manuel.jimenez@upm.es (M.J.-H.); enriquejavier.gomez@upm.es (E.J.G.); 2 Institut Guttmann, Institut Universitari de Neurorehabilitació adscrit a la UAB, 08916 Badalona, Spain; gcattaneo@guttmann.com (G.C.); aroca@guttmann.com (A.R.); jmtormos@guttmann.com (J.M.T.-M.); dbartres@ub.edu (D.B.-F.); apleone@hsl.harvard.edu (Á.P.-L.); 3 Department of Medicine, Universitat Autònoma de Barcelona, 08035 Bellaterra, Spain; 4 Centro de Investigación Biomédica en Red, Biomateriales y Nanomedicina (CIBER-BBN), 28029 Madrid, Spain; 5 Department of Neurology, University of Miami Miller School of Medicine, Miami, FL 33136, USA; jgomes@linus.health; 6 Linus Health, Boston, MA 02451, USA; 7 Departament de Medicina, Facultat de Medicina i Ciències de la Salut i Institut de Neurociències, Universitat de Barcelona, 08036 Barcelona, Spain; 8 Hinda and Arthur Marcus Institute for Aging Research, Hebrew SeniorLife, Boston, MA 02131, USA; 9 Deanna and Sidney Wolk Center for Memory Health, Hebrew SeniorLife, Boston, MA 02131, USA; 10 Department of Neurology, Harvard Medical School, Boston, MA 02115, USA

**Keywords:** mHealth, brain health, decision support, intervention, monitoring, coaching, healthy lifestyles

## Abstract

Brain Health is defined as the development and preservation of optimal brain integrity and neural network functioning for a given age. Recent studies have related healthy habits with better maintenance of brain health across the lifespan. As a part of the Barcelona Brain Health Initiative (BBHI), a mHealth platform has been developed with the purpose of helping people to improve and monitor their healthy habits, facilitating the delivery of health coaching strategies. A decision support system (DSS), named Intelligent Coaching Assistant (ICA), has been developed to ease the work of professional brain health coaches, helping them design and monitor adherence to multidomain interventions in a more efficient manner. Personalized recommendations are based on users’ current healthy habits, individual preferences, and motivational aspects. Taking these inputs, an initial user profile is defined, and the ICA applies an algorithm for determining the most suitable personalized intervention plan. An initial validation has been done focusing on assessing the feasibility and usability of the solution, involving 20 participants for three weeks. We conclude that this kind of technology-based intervention is feasible and implementable in real-world settings. Importantly, the personalized intervention proposal generated by the DSS is feasible and its acceptability and usability are high.

## 1. Introduction

Brain Health is defined [1] as “the development and preservation of optimal brain integrity and neural network functioning for a given age”. To determine which factors exert the highest influence on brain health preservation, the Barcelona Brain Health Initiative [1] (BBHI) is carrying out a prospective, population-based, longitudinal study with more than 5500 volunteers between the ages of 40 and 65 years free from any neurological or psychiatric disease. BBHI defines seven domains related to brain health (brain health pillars): physical exercise, sleep, cognitive activity, nutrition, socialization, vital plan, and general health. Although behaviors related to these pillars are demonstrated to influence brain health, their relative importance is likely to differ, both at a group level and an individual level. For example, in the ongoing BBHI longitudinal study, changes in a vital plan at follow-up, as well as gender, sleep quality, and sense of coherence (a cognitive component consisting of the ability to make sense of our experiences) at baseline were shown to be significant risk factors for the onset of new diagnoses [2]. Furthermore, on an individual basis, certain aspects may be more salient or actionable to each person; for example, managing sleep disturbances may be a priority to some, and being more active a priority for others. Conversely, someone who sustains healthy eating habits and has strong social support may still take active steps to improve their brain health by being purposeful regarding their vital plan, and improving sleep hygiene.

As a nested study within the whole initiative, the Intelligent Brain Coaching (iBC) project [3] is developing different tools to help individuals improve their healthy habits in the form of lifestyle coaching and monitoring, with the ultimate goal of improving their overall brain health. As the first step in this effort, we carried out a systematic review of the state of the art for monitoring technologies applied to brain health [4]. We concluded that most valuable studies were done in older adults and frequently focused on just a single domain like nutrition or physical exercise rather than taking a multidomain approach. This work also analyzed different kinds of intervention programs and identified a key knowledge gap in the use of automatization and artificial intelligence processes to support lifestyle interventions for the promotion of brain health in aging and older adults. Similarly, a more recent review by Markert et al. [5] concludes that to date there are not many decision support systems (DSS) to assist active and healthy aging. Nonetheless, a few examples of the application of DSS to promote brain health can be found in literature. For example, Moschonis et al. [6] developed a DSS to improve diet habits, treat and prevent childhood obesity and showed that the intervention group improved their habits whereas the control group (without the benefit of the DSS) increased their body mass index. Another example is the implementation of a DSS for personalized coaching in the aging population by Orte et al. [7], though this has not been yet been validated.

In this article, we present a decision support algorithm for coaching and promoting healthy habits related to brain health. This decision support algorithm is an important component of a mHealth platform developed with the specific aim of implementing a multidomain intervention to optimize lifestyle habits. This platform consists of two main components: (i) a web portal for the coach to supervise the intervention program, and (ii) a mobile application where users receive personalized recommendations and pieces of advice in the form of “knowledge pills” to help them improve their habits in the above-mentioned seven pillars of brain health. This mobile app also includes a specific module for cognitive training, with a set of 12 computerized tasks that train core cognitive functions, such as attention, memory, or executive functions. The cognitive training module has been specifically designed for mobile devices, evolving existing tasks from the Guttmann Neuro Personal Trainer (GNPT) platform [8]. The app also monitors daily physical activity and sleep quality by integrating a commercially available wearable device [9] and includes a questionnaire for monitoring adherence to the Mediterranean diet on a weekly basis [10].

The main purpose of this work is to present and discuss the results from an initial validation focused on the feasibility and usability of the mHealth platform for coaching and promoting healthy habits related to brain health, together with some adherence and performance indicators. The manuscript is organized as follows: first, the materials and methods section goes over all the needed concepts and definitions, as well as a detailed description of the design of the decision support system and implementation of the algorithm that automatically selects and configure the most suitable personalized recommendations for the user. Then, the results section describes the metrics and performance indicators for the usability and feasibility study, distinguishing the results from the previous technical validation pilot carried out. Finally, the last section covers the discussion of the results, highlighting the identified limitations and presenting the main conclusions and future work foreseen.

## 2. Materials and Methods

The Intelligent Coaching Assistant (ICA) has been developed following the stages described below. The intervention process starts with a screening phase where validated online questionnaires are completed by the users. This stage is aimed at gathering critical baseline information about each user’s lifestyle regarding the seven identified brain health pillars. In addition, we obtain information about subjective health perception, including cognitive complaints and quality of life measures [1]. Taking this input, the initially defined service model includes interactions between the user and a professional with the role of health coach with the goal of determining preferences and motivational aspects that are relevant for personalizing interventions. This interaction is foreseen to happen in different ways, including face-to-face visits or remote (video or phone calls) tele-health sessions. However, most of the interaction is conceived to happen through the mHealth solution, by sending messages through the mobile app that include recommendations and pieces of advice regarding healthy habits. These pieces of advice aim to go beyond simple, generic recommendations and thus are defined as prescriptions of “knowledge pills” delivered to the user through the notification system of the smartphone. A set of knowledge pills for each brain health pillar has been designed by professionals from the Institute Guttmann and the BBHI research team, an interdisciplinary team including neuropsychologists, neurologists, psychologists, physiotherapists, and physical trainers. Besides, thanks to the collaboration with members of the Scientific Advisory Board [11] and partner institutions [12] of the BBHI, other knowledge areas have been extended to address all defined pillars. These knowledge pills have been defined based on available scientific evidence regarding the healthy habits reported to have the greatest impact on brain health. The only brain health pillar defined in BBHI that has not been covered in this solution is the “General health” one, since this pillar is conceived to be the consequence or final goal after properly managing healthy lifestyles in the other six ones, which are the focus of the solution: physical exercise, cognitive, sleep, nutrition, socialization, and vital plan.

From the available library of knowledge pills, the coach selects the most suitable for each user, thus, effectively compiling a personalized therapeutic regimen. Along the intervention process, further information from app monitoring is available to the coach to reconsider, update and modify these personalized recommendations. Before providing more details, a set of key concepts and definitions are explained next.

### 2.1. Intervention Design and Goals

The mHealth platform, where the ICA is integrated, aims at enabling the monitoring and supervision of multidomain interventions. For this specific initial usability and feasibility pilot study of the ICA, a multidomain intervention has been defined, including the following specific-domain goals:

- Cognitive activity: Computerized Cognitive Training (CCT) involves the repeated practice of a set of tasks that are structured and standardized, which have been designed to train one or more cognitive skills. Following the evidence from the literature [13,14], participants were asked to do at least 60 min of cognitive training per week, divided into 3 sessions on different days, with a duration of at least 20 min per session. For this purpose, the mHealth solution integrates a specific module for cognitive training, consisting of 12 computerized tasks.
- Nutrition: participants will be asked to follow Mediterranean diet indications and reduce the consumption of salt, which according to literature [15,16] prevents cognition decline. The common general guidelines of the Mediterranean Diet include the use of olive oil (for both cooking and season dishes), consumption of ≥2 daily servings of vegetables, consumption of ≥2–3 daily servings of fresh fruits, ≥3 weekly servings of legumes, ≥3 weekly servings of fish or seafood, ≥3 weekly servings of nuts or seeds; select white meats instead of red meats or processed meats (burgers, sausages); cook twice a week with tomato, garlic and onion, and dress vegetables, pasta, rice and other dishes with tomato and garlic, reduce and if possible eliminate the intake of cream, butter, margarine, cold meat, pâté, duck, carbonated and/or sugared beverages, pastries, industrial bakery products. Participants will be suggested to do 2 main meals, seated at a table and lasting more than 20 min per day, should be eaten. In order to monitor this, the system includes a questionnaire for monitoring adherence to the Mediterranean diet on a weekly basis [10].
- Sleep: participants will be asked to follow healthy sleep habits. Specifically, participants will be encouraged to follow the recommendations for healthy sleep proposed by the Global Council for Brain Health [17], being the main recommendation to get about 7–8 h of sleep in a 24-h period. This domain will be monitored by the wearable device integrated, measuring the sleep time and quality per day.
- Physical exercise: participants will be asked to follow WHO recommendations [18] and do 2 to 3 sessions per week of moderate to intense physical activity, of a duration of 30 to 60 min per session. As well as sleep, this domain will be monitored by the wearable device integrated, which measures not only the time expended doing exercise but also the intensity thanks to the heart rate monitoring device.

Apart from this intervention proposal, participants will receive a set of personalized advice for the six domains (physical exercise, cognitive, nutrition, sleep, socialization, and vital plan), which are the basis of the ICA decision support tool presented in this work. These personalized advice are delivered to the user in form of brain health knowledge pills, as it is explained in next Section 2.2.

### 2.2. Brain Health Knowledge Pills

A knowledge pill is a short tip or piece of advice focused on addressing an important aspect or overcoming a lack of knowledge regarding activities that promote brain health. The name “pill” is inspired by its similarities to medications traditionally used in clinical practice, where a clinician prescribes specific substances—prepared in the form of a pill—for treating a certain diagnosis or condition. In our context, brain health pills are delivered to users who want to adopt or change a habit to maintain or improve their health. Therefore, brain health pills can include educational and motivational interventions, as well as multimodal approaches to assess, monitor, support, and promote brain health-promoting attitudes and behaviors. We have defined the concept of Master Pill, which describes the basic essence of the pill in a generic manner, while the Custom Pill is the final, personalized version of the Master Pill, adjusted to fit specific needs and preferences for a given user. To get to the final Custom Pill, the Master Pill receives a layer of personalization based on the target final user. An example of Master Pill would be “Increase the amount of daily exercise” which for a specific user who likes outdoor sports, nature, and works from 9 a.m. to 6 p.m. could be personalized into the following Custom Pill: “Get up half an hour earlier and go for a relaxing walk in the park before you start your workday”. It is noteworthy the system does not categorize pills into a single domain, but rather is designed to work with multidomain pills, so they can be associated to different domains each one with a specific percentage. For example, pill “Volunteer in the community” is associated both with the “Vital plan” pillar and “Socialization” pillar.

### 2.3. Algorithm Design

The inputs of the algorithm come from 3 sources, as shown in Table 1. The first source is the initial questionnaire (Q1) built from a set of validated questionaries, gathering information about current lifestyles and self-perceived health status [1,2] for all the domains defined in the system. The second source is the preferences questionnaire filled in by the user the first time s/he logs in the app. However, since the preferences can change over time, it is possible for the user to modify the initial selections. The last source of information is related to the feedback from the app itself, where there is information about pills feedback and monitored data from all the domains described before.

The process defined for the ICA has been designed to work on a weekly basis, making an intervention proposal for the next seven days, taking into account the information monitored and the user feedback given through the app, so it learns from previous user’s performance and opinion to increase the personalization. It is important to highlight that the ICA, as a DSS, generates an intervention proposal that the professional can modify and fine-tune afterward.

**Figure 1:**
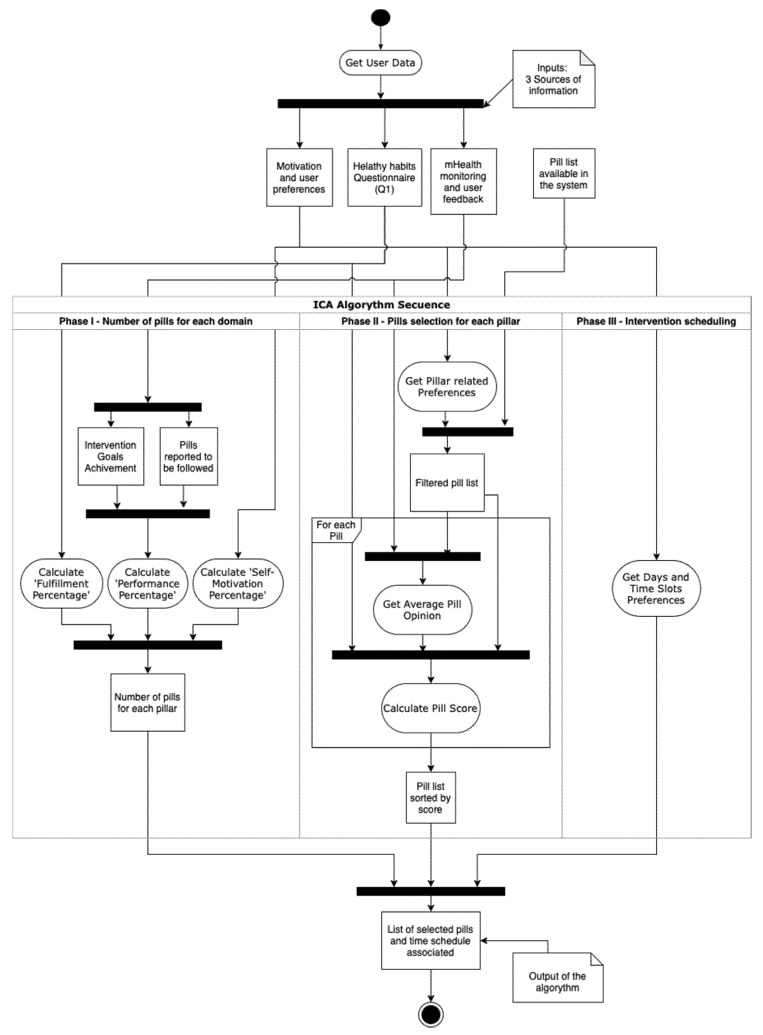
UML Activity Diagram illustrating the process followed by the algorithm for completing the intervention proposal.

A summary of the process can be seen in Figure 1. The algorithm is divided into the three phases described in the next subsections.

#### 2.3.1. Phase I. Number of Pills for Each Domain

Based on the favorite days and slots preferred by the user from the motivation and user’s preferences data source, the ICA estimates the total number of pills that will be selected for the next 7-days period. The length of the period and the number of slots in each day can be modified. Similarly, the main drivers of the number of pills suggested for each user can also be modified. For our pilot study, the number of time slots selected by the user was used for determining the maximum number of pills, having three available options for a day (mornings, afternoons, evenings).

**Figure 2:**
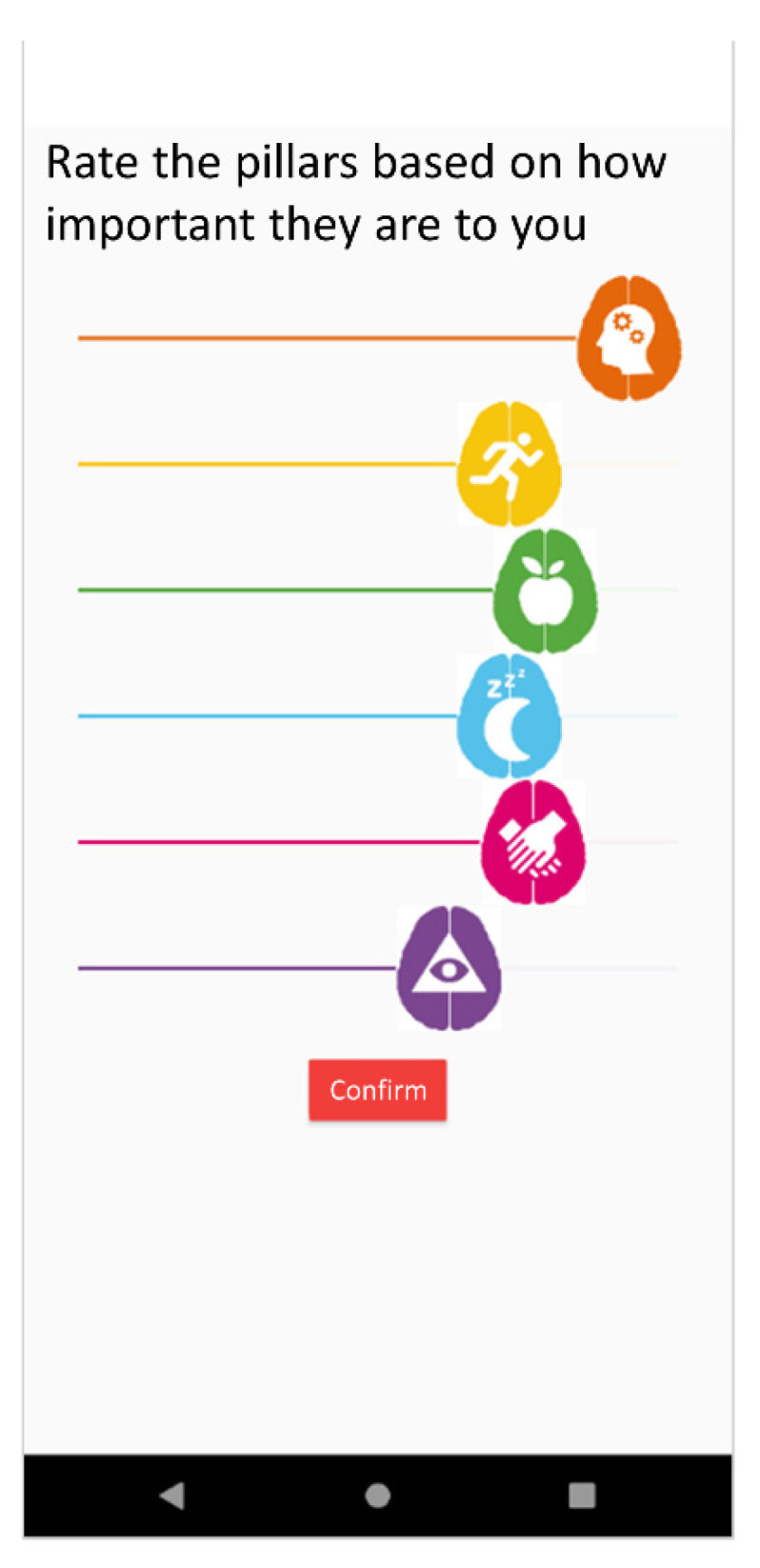
App screen where users rate their self-motivation percentage for each domain.

At present, the ICA defines the number of pills to be related to each pillar as follows. However, it is noteworthy that the specific rationale to define the selection of pills can certainly be modified. and other criteria can be applied, for example, to target specific brain health pillars. It is also possible to establish a feedback loop such that the method for selection of the number of pills and the brain health pillars to be targeted could evolve over time. In any case, the initial method that has been implemented in our pilot study considers the following:

- Fulfillment percentage. Based on the information gathered from the healthy habits initial questionnaire (Q1), the system determines a score for each brain health pillar and, with that, a presumed ‘need’ for each user. For example, for the nutrition pillar, the system uses the 14-item Mediterranean adherence questionnaire [19], which defines a response criterion for giving 1 point to each answer. So, the maximum score that can be obtained by a user is 14. The maximum score obtainable for each pillar is defined as 100% fulfillment for that domain and implies no need to further focus on the promotion of that domain. If the score is lower than the maximal score obtainable for a given pillar, the ‘fulfillment percentage’ is calculated. The lower the fulfillment percentage the higher the priority to prescribe brain health pills for a given brain health pillar.
- Performance percentage. It reflects the degree of work performed by the user in the app. It is calculated as the average completion of tasks (compliance with pills) performed in previous days up to the last 4 weeks, which corresponds to the mHealth monitoring and user feedback data source. If there is no information available, it is taken as zero. The performance percentage (compliance with pills) considers the following two issues: (1) The percentage of pills a given user has adhered to for a specific pillar determines 25% of the performance percentage; (2) The remaining 75% is determined by the specific tasks for each pillar. For example, sleeping an average between 6 and 8 h daily for sleep pillar, or doing between 30 and 40 min of aerobic exercise twice a week.
- Need percentage. Defined as the inverse of the previous fulfillment percentage and calculated for each domain with Equation (1).
  (1) Need%=100−Fulfillment%
- Self-motivation percentage. It reflects the preferences expressed by a given user regarding the desire to work more on one pillar or other in the motivation and user preferences data source. This is reported by the user upon signing up for the app but can be modified subsequently. This uses a slider to give a response to the question “Rate the pillars based on how important they are to you?” as shown in Figure 2.
- Final percentage. It represents the overall importance determined by the system for each pillar for a given user. It is calculated with Equation (2). In this equation, the percentages of pillars that are more necessary for a given user either based on need or based on preference and motivation are added, while the percentages of pillars most worked on over in previous days up to 4 weeks are subtracted.
  (2) Final% =Need%+ Selfmotivation% −Performance%
  Once this percentage is calculated for each domain, the number of pills to be selected for each domain is calculated with Equation (3).
  (3) $$n\;\mathrm{of}\;\mathrm{pills}\;\mathrm{for}\;\mathrm{domain}=\frac{\mathrm{Domain}\;\mathrm{Final}\%\;}{\sum \mathrm{Domain}\;\mathrm{Final}\%\;}\times \mathrm{Total}\;n\;\mathrm{of}\;\mathrm{pills}$$
  NOTE: In case that every percentage is zero, pills will be distributed equally in each pillar to avoid zero division.

#### 2.3.2. Phase II. Pills Selection for Each Pillar

Once the number (amount) of pills for each pillar has been set, in this second phase, the algorithm selects specific pills from those available in the system.

First, pills are filtered based on user’s preferences. For example, if user’s preference for diet is vegan, all pills related to meat or fish consumption will be discarded.

Then, each pill receives a suitability score based on its affinity to the user. Equation (4) shows how the suitability score of a pill is calculated for each user. This score goes from 0 to 10 and is based on the Q1 questionnaire responses. Each pill is affected by specific questions and its answers. A weight parameter is assigned to the relation between a pill and a question that expresses its strength. This weight parameter goes from 0 to 5, but not all the pills have the same number of questions related. To normalize the score, the weight parameter obtained from each user is divided by the maximum weight parameter obtainable for that pill and then multiplied by ten.

(4) $$\mathrm{Suitability}\;\mathrm{score}\;=\frac{\mathrm{Weight}\;\mathrm{parameter}\;\mathrm{obtained}}{\mathrm{Maximum}\;\mathrm{weight}\;\mathrm{parameter}\;\mathrm{obtainable}}\times 10\;$$

**Figure 3:**
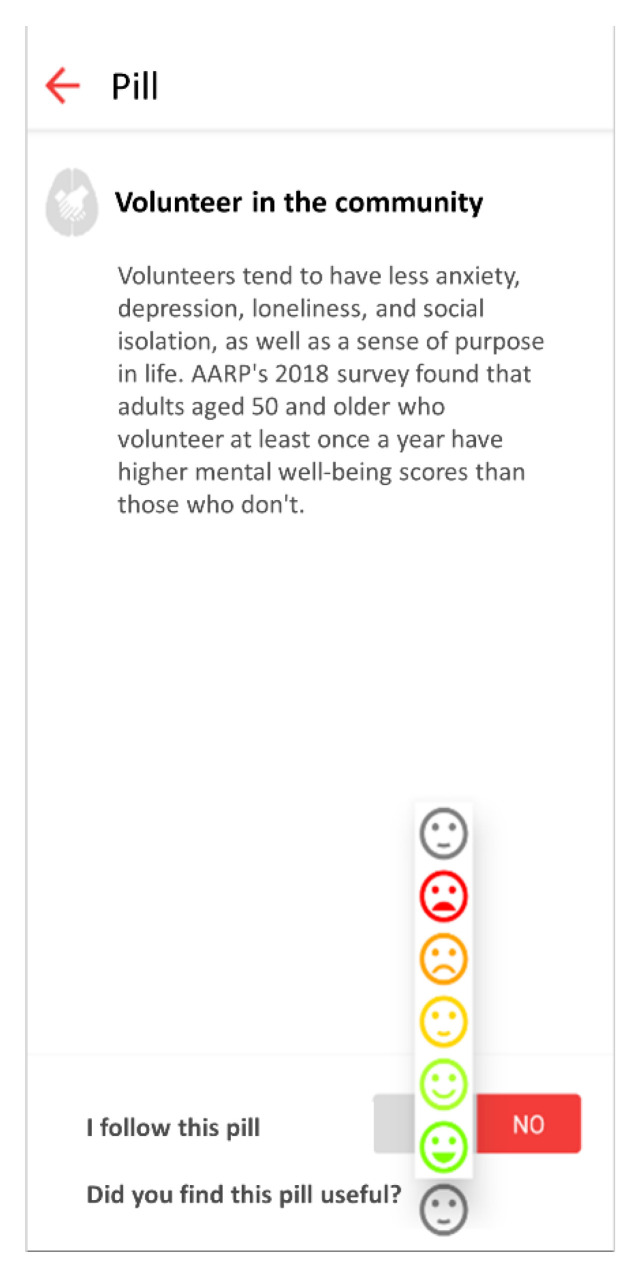
This figure shows the graphical Likert scale to evaluate received pills.

The mobile application allows users to provide feedback on each received knowledge pill with a graphical Likert scale from 1 (terrible) to 5 (excellent), as shown in Figure 3.

Table 2 shows how the score calculated to a pill is modified depending on the average value of the feedback received for that pill by other users to penalize those pills with the lowest scores.

Then, applying content-based filtering to the resultant score, the final score for each pill is obtained. To avoid repeating the delivery of a pill to a user when an opinion has been already done, the system penalizes the score for that specific pill as specified in Table 3.

#### 2.3.3. Phase III. Intervention Scheduling

This is the final phase of the ICA algorithm. First, for each domain, pills with greater scores are selected until the number of pills for each pillar is reached, or there are no more available pills for that pillar.

When the final selection of pills is completed, it is distributed in the following 7 days. Each day is divided into three-day slots: morning, afternoon, and evening. Pills scheduled in the morning gap are sent at 9 a.m., 5 p.m. for the afternoon, and 9 p.m. for evenings. The process starts by randomly distribute pills into the days and slots previously selected by the user in the preferences configuration. Then, if each preferred slot contains more than two pills and there are still pills pending to be scheduled, these remaining pills are randomly assigned to the rest of the free slots. Since the maximum number of pills is calculated at the beginning of the process, this second iteration should not take place except for rare occasions where users have restricted too much their preferred slots and it is performed to avoid low significant interventions.

### 2.4. ICA Integration in the mHealth Platform

The ICA integration in the BBHI mHealth platform has been designed applying UX and usability basis. The main purpose of this design is to simplify and make the use of this tool as intuitive as for the professional coach that manages and supervises the healthy habits program.

Three key requirements were defined:

1. Intervention schedule generated must be approved by the coach and cannot be accidentally validated.
2. Personal information taken as input for the algorithm (healthy habits monitor, personal preferences, motivation, and performance monitored) must be accessible to the coach to ease the decision-making.
3. Algorithm must be transparent to the coach to not persuade him/her of his criteria.

**Figure 4:**
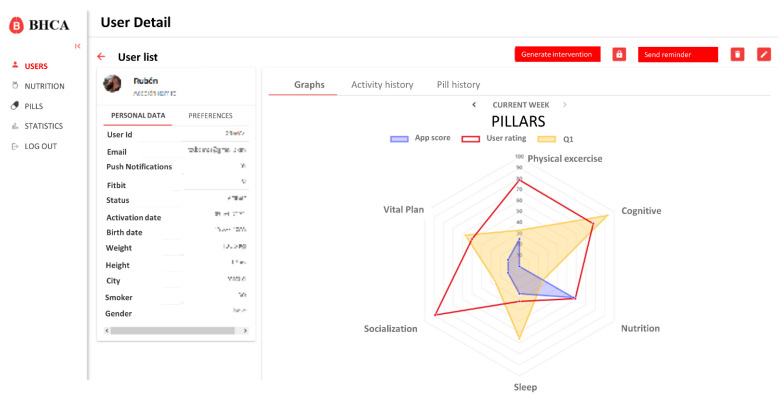
Main page with detailed information about a user. On the left side there are personal data and preferences selected by the user. On the right side there is a graphical representation of his/her pillars scores. Personal data has been removed.

Based on these requirements, the integration only affects the user profile and detail data. On this view, a new button to generate the intervention and access to the scheduling view was created. Also, different tabs to visualize the new data were added. These changes are shown in Figure 4.

**Figure 5:**
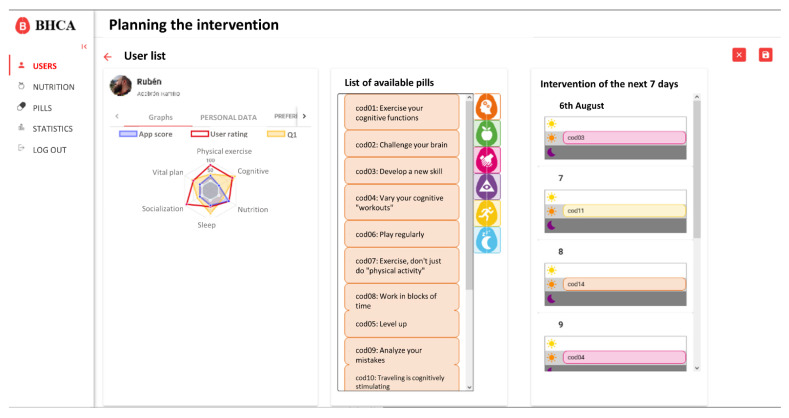
Main page of intervention generation. The left side displays the personal data of the user as well as his scores and preferences. In the middle section displays the list of available pills in the system. The right side displays the schedule scheme for the next seven days divided in morning, afternoon, and evening. After saving the schedule the web application redirects to the detail user web (Figure 4) and the new pills added on the schedule can be seen on the detail of intervention (Figure 6).

On the intervention scheduling view, the coach has access to specific data of the user, as well as the list of available pills in the system and the schedule for the next seven days (Figure 5). Initially, this view will be automatically populated with the proposal generated by the algorithm, but the coach will be able to easily modify it by dragging and dropping the pills from the list of available pills to the schedule and vice versa.

**Figure 6:**
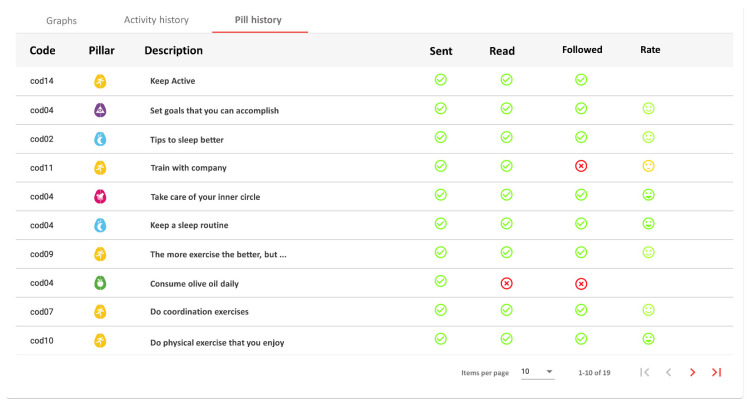
Detail of an intervention. The coach can see the pill sent, and if the user has read, followed and given his opinion.

Both the automatically generated intervention and the final intervention approved by the coach will be saved to study the divergences in the future. The pills arranged in the intervention are visible in the application, as shown in Figure 6.

### 2.5. Validation Plan Design

The main objective of the validation presented in this work is to measure the usability of the present solution and the feasibility of intervention programs based on it. To achieve this, volunteers were selected from the already established BBHI cohort [1] (men and women aged 40 to 65 years old and without a diagnosis of neurological disease or psychiatric condition). Users were asked to fill out two questionnaires about usability and perceived personalization. The usability questionnaire selected is the System Usability Scale (SUS) [20]. Its results were analyzed to determine the percentile, acceptance, and Net Promoter Score (NPS).

Also, the monitoring data obtained from users were analyzed to determine the adherence to the intervention proposed. Physical activity and sleep monitored with the Fitbit wearable, as well as the performance in cognitive training tasks, were the main sources of information used to determine adherence. Finally, the pills and their reception by users were analyzed, considering if a user read a received pill, if he/she reported following the advice, and the opinion/feedback provided upon reception of a pill.

## 3. Results

The results presented here are divided into two subsections. The first is related to the initial technical validation pilot, while the second one is related to the feasibility and usability.

### 3.1. Results of Technical Validation Pilot

Six users (age 30 ± 10.35 years, female 33, 34%) were selected for an initial technical validation pilot. They were not part of the BBHI cohort. The users were asked to interact with the application for three weeks. The main goal of this study was to debug and evaluate the functionality and general usability of the application.

Some minor issues and bugs were reported, mainly related to the notification service and some issues with the graphical representation of the percentages of the fulfillment of some pillars.

### 3.2. Results of Feasibility and Usability Pilot

After resolving the errors and bugs reported on the technical validation, a feasibility and usability pilot was carried out involving 14 participants from the BBHI cohort (age 55.3 ± 7.2 years, female 50%). They reported average skills on computer usage and interacted with the application for three weeks. A Fitbit Charge 4 device was lent to each participant during the pilot.

Users were asked to use the app normally, synchronizing their Fitbit device and filling the nutrition questionnaire regularly. The nutrition questionnaire was filled in between one and two times per user on average. Users were asked to register at least three physical activity sessions per week of moderate to vigorous physical activity. Regarding cognitive training, users were asked to perform three sessions per week of at least 20 min of training. We found that they completed the objective on average at 302% and 167%, respectively, as shown in Table 4. These higher numbers are possible because 85% of users continued to use the app and to register activities after the pilot trial period ended. Additionally, users were asked to sleep at least 7 h per day. They fulfilled this objective 47.28% of the days on average.

As it can be seen in Table 5, users have read 88.09% of the received pills, reported to follow the received advice for 65.86% of the cases, and gave their opinion for 50.18% of them. The average opinion on pills is 4.15/5. It should be noted that one of the users neither read nor reported to follow none of the pills received.

**Figure 7:**
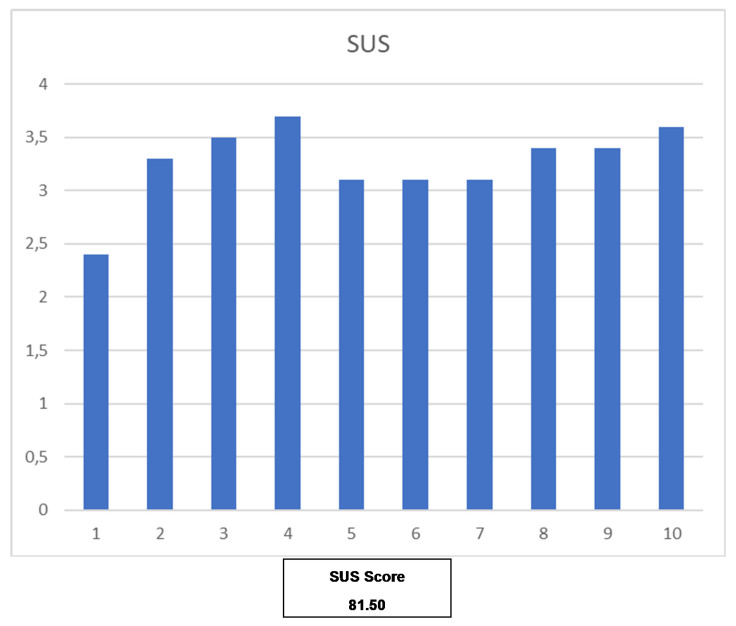
Detail score of each question on SUS and the total score. Score for odd questions is calculated as “Response—1” whereas score for even questions is calculated as “5—Response”. Therefore, each question score goes from 0 to 4. The final SUS Score is calculated adding all the questions scores and multiplying them by 2.5 [21].

Table 6 shows the summary of the SUS questionnaire [20]. These questionnaires were completed by 85% of users (*n* = 12). As it is shown in Figure 7, the score obtained in the SUS questionnaire is 81.5, which is associated with grade A and adjective excellent. It is considered promoter in NPS, and it is in percentile 90–95 [21].

**Figure 8:**
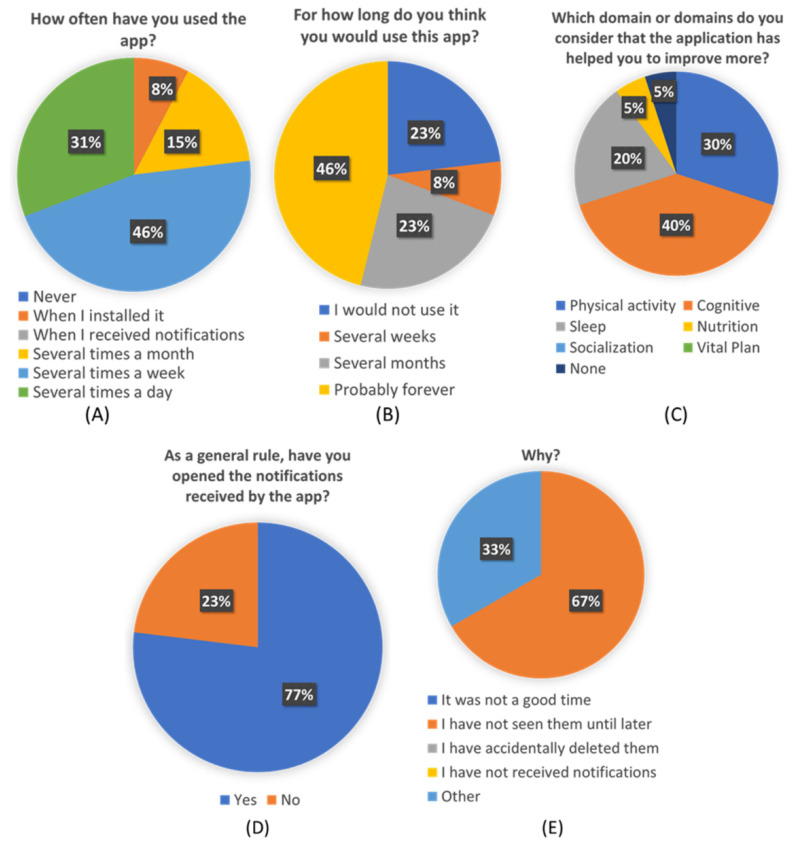
Detail score of each question “Personalization Perceived Questionnaire” for the questions 9 to 12. (**A**): Score of question 9: “How often have you used the app?”; (**B**): Score of question 10: “For how long do you think you would use this app?”; (**C**): Score of question 11: “Which domain or domains do you consider that the application has helped you to improve more?”; (**D**): Score of question 12a: “As a general rule, have you opened the notifications received by the app?”; (**E**): Score of question 12b: “Why?”.

In order to complete this assessment and look into specific aspects related to user experience, a custom questionnaire was designed and named “Personalization Perceived Questionnaire”. This questionnaire consists of six questions with Likert 5 scale response kind, and five questions with given options. In Table 7 questions that were answered by a Likert scale are shown. In Figure 8 questions which other kinds of answers that are different from the Likert scale are shown.

## 4. Discussion

In spring 2020, the arrival of the COVID-19 pandemic had a great impact on every research activity related to projects like BBHI, and specifically on those activities requiring face-to-face interactions like the multimodal intervention validation presented in this study. Then, we turned the crisis into an opportunity of improving the above presented initially defined process, to make it less dependent on presential interactions, and designed a Decision Support System (DSS) to help professional coaches to deliver their services through the mHealth platform on a more efficient manner. This DSS was inspired by the success case of a previously DSS implemented in the GNPT platform [22], which has been widely used for the last 10 years in real clinical settings.

After the feasibility and usability pilots have been carried out, we can conclude that the results obtained are very promising. The mHealth platform is perceived to be useful, usable and the adherence monitored shows that this kind of technology-based multidomain intervention is feasible. Furthermore, the collected data, thanks to the app itself and the integrated wearable device, is enough for monitoring basic aspects of daily routines and study their possible relation to brain health. Based on other studies as Hawley-Hague et al. [23] and Kivipelto et al. [24], these high percentages on objectives achievement, reflected in the results, seem to indicate a high level of adherence in terms of interest, perception, and grade of fulfillment. However, looking at the initial pilot presented and considering the early stage of validation of the mHealth solution, some important limitations, and future works have been identified, which are discussed next.

The usability evaluation results are promising and report a SUS score of 81.5, which situates the mHealth solution in the second-highest percentile (90–95) [20]. From this, we can conclude that the users can be perceived as potential ‘Promoters’ of the solution based on the Net Promoter Score, while the general mHealth solution obtains the adjective ‘Excellent’. However, due to the size of the population participating in the study, caution is required when interpreting these results, and more usability evaluations will be covered by further pilots. Nevertheless, these results are encouraging and demonstrate that the basis of the mHealth solution is perceived as usable.

From the custom-created “Personalization Perceived Questionnaire”, some interesting conclusions can be extracted. More than a third of participants think they would use this app forever. And more than a half will use it at least during several months. Almost three-quarters of the participants used the app several times a week, and more than 80% of them found the application useful in different ways.

Users have also reflected that the received pills were accurate to their needs and their profiles and preferences. This seems to point that de ICA algorithm is working correctly and efficiently. The average score of 4.1 given to the pills seems to indicate that users are also satisfied with the content of pills and find them interesting and useful to learn about healthy habits and brain health. Although Figure 8C shows that not all the pillars are equally perceived by users, which also shows an opportunity to refine the overall recommendations and focus on the pillars that were less identified to be addressed, such as vital plan, which seems to be critical for overall brain health.

Another interesting result is the difference in pills interaction among users, according to the results shown in Table 6. Whereas most of the users received between three and six pills, three users received more than 10, and two users received two. Considering that the number of pills received is fully dependent on the user preferences for pill scheduling, it could mean that users with more than 10 pills had a high initial motivation, while users with less than three could have an especially low initial motivation. However, users that received more than 10 did not have an especially high follow rate or opinion rate. On the other hand, one of the users with two pills did not read, follow, or rate any pill. Since no technical problem was reported, this seems to indicate a total lack of adherence or motivation.

This shows that our system has still some limitations and needed improvements. Specifically, this problem of a persistent lack of adherence is expected to be resolved by the professional coach personally. The system provides the professional coach effective and quick information about user follow-up (see Figure 5), being able to early identify lack of adherence, contacting the person to identify the reasons, and try to increase motivation.

Another important identified limitation is the lack of an age-based adjustment on the DSS on pill selection. In this regard, more research is needed in order to identify personal preferences and/or habits-related aspects that can be affected by age, and that can be reflected as customizations of *Master pills* to increase personalization.

It must be also clarified that the mHealth solution presented is on a living process, to ensure that the list of available pills is up to date to the last evidence published in the literature. So, the solution has been designed having in mind the need of updating and adding pills, and professionals can easily do it through the web portal.

More evaluation pilots will be carried out in other studies to complement the results presented in this work. Results from activity and sleep monitoring are promising. Most of the users use the Fitbit device daily even over the pilot duration, although some of the users report being uncomfortable, especially during sleep time, with the wearable or did not use it. In addition, data collected from Fitbit API can be a limitation because the minutes in moderate-high intensity exercise are calculated by Fitbit’s algorithms and could not be very accurate. Based on our experience, this kind of commercial device works much better when the user actively starts the registration of activity, compared to when the device determines an activity on an automatic basis. As a lesson learned for future pilots, more emphasis should be placed on asking the person to actively record activities via the wearable interface, avoiding automatic activity detection as much as possible. To improve this limitation, we plan to develop a middleware that enables the possibility of connecting other commercial devices as well as other monitoring independent systems.

The main goal of both BBHI and iBC projects is to enable the transfer of their positive results to real-world settings. This mHealth application could provide an effective way to improve the effectiveness of lifestyle interventions to increase the adherence of people to healthy habits and maintain behavioral changes over longer periods, helping to prevent the decline of brain health as well as other diseases related to lifestyles. To achieve this goal, the next step is to carry out a randomized control study to evaluate healthy habits adherence and its impact on users’ brain health, including an evaluation pre and post-intervention. This study will put the focus also in evaluating the impact on efficiency about how professionals deliver brain health coaching services over innovative solutions.

## Figures and Tables

**Table 1:** This table summarizes the different sources of information taken as input sources by the ICA algorithm.

Initial Questionnaire (Q1)	Motivation and User Preferences	mHealth Monitoring and User Feedback
Medical history	Motivation	Sleep monitoring data
Socio-demographic data	Allergies and intolerances	Physical activity monitoring data
Nutritional habits self-awareness	Outdoor/indoor sport preference	Nutrition questionnaire
Physical Activity self-awareness	Group/Solo activities preference	Cognitive tasks
Physical status self-awareness	Special sleep conditions (Night work, nap, etc.)	Pills feedback
Goals and objectives in life	Pills schedule preferences	Pills follow up
Personality and way of facing problems		
Socialization habits self-awareness		
Social self-awareness		
Sleep habits self-awareness		
Cognitive status self-awareness		
Cognitive reserve		

**Table 2:** This table presents the score modifier associated to the pill average opinion value.

Opinion Value	Score Modifier
5, Excellent	+1.0
4, Good	+0.5
3, Normal	0
2, Bad	−0.5
1, Terrible	−1.0

**Table 3:** This table presents the score penalization associated to the pill opinion value from the same user receiving the pill.

Opinion Value	Score Modifier
5, Excellent	−1.0
4, Good	−2.0
3, Normal	−3.0
2, Bad	−4.0
1, Terrible	−5.0

**Table 4:** This table presents the results of exercise domain, sleep domain and cognitive training domain. Exercise domain is divided in two main metrics, intense exercise and more than 10,000 steps.

Users	Moderate-Intense Exercise			>10,000 Steps		Sleep		Cognitive Training		
	Days	% Over Objective	% Over Total	Days	%	Days	%	Days	% Over Objective	% Over Total
User 1	22	244%	105%	21	100%	3	14%	8	89%	38%
User 2	37	411%	176%	36	171%	25	119%	36	400%	171%
User 3	26	289%	124%	24	114%	12	57%	21	233%	100%
User 4	25	278%	119%	24	114%	25	119%	8	89%	38%
User 5	29	322%	138%	27	129%	4	19%	26	289%	124%
User 6	16	178%	76%	15	71%	1	5%	9	100%	43%
User 7	6	67%	29%	5	24%	15	71%	10	111%	48%
User 8	45	500%	214%	43	205%	4	19%	9	100%	43%
User 9	28	311%	133%	26	124%	12	57%	19	211%	90%
User 10	43	478%	205%	41	195%	18	86%	4	44%	19%
User 11	36	400%	171%	34	162%	5	24%	25	278%	119%
User 12	29	322%	138%	27	129%	3	14%	16	178%	76%
User 13	37	411%	176%	35	167%	12	57%	14	156%	67%
User 14	2	22%	10%	1	5%	0	0%	5	56%	24%
Mean	27.21	302%	130%	25.64	122%	22.64	47.28%	15	167%	71%

**Table 5:** This table presents the results users feedback from pills received.

User	Pills				
	Received	Average Rate	% Rated	% Followed	% Read
User 1	17	3	5.88	88.23	100
User 2	3	5	100	100	100
User 3	12	3.71	58.33	83.33	100
User 4	3	5	66.67	33.33	66.67
User 5	3	5	33.33	33.33	100
User 6	4	3	50	50	100
User 7	2	-	0	0	0
User 8	31	5	96.77	83.87	100
User 9	3	-	0	66.67	100
User 10	4	4	25	50	100
User 11	2	4	50	100	100
User 12	6	3.67	50	100	100
User 13	4	4.5	100	100	100
User 14	3	4	66.67	33.33	66.67
Mean	-	4.15	50.18	65.86	88.09

**Table 6:** This table presents the results of questions from SUS usability questionnaire.

	Totally Disagree	Disagree	Indifferent	Agree	Totally Agree
1. I think I would like to use app “BHCA” frequently	1	2	1	4	2
2. I found the “BHCA” app unnecessarily complex	6	2	1	1	0
3. I thought the app was easy to use	0	1	0	2	7
4. I think that I would need the support of a technical person to be able to use this app	7	3	0	0	0
5. I found the various functions in this app were well integrated	0	1	0	6	3
6. I thought there was too much inconsistency in this app	5	2	2	1	0
7. I would imagine that most people would learn to use this system very quickly	0	1	1	4	4
8. I found the system very cumbersome to use	6	2	2	0	0
9. I felt very confident using the system	0	1	0	3	6
10. I needed to learn a lot of things before I could get going with this app	7	2	1	0	0

**Table 7:** This table presents the results of questions from “Personalization Perceived Questionnaire”.

	Totally Disagree	Disagree	Indifferent	Agree	Totally Agree
1. I am used to technology and mobile phones	0	0	4	4	5
2. Pills received fit my profile and preferences	1	2	3	5	2
3. I thought the app adapted my needs, preferences and motivations	1	0	6	3	3
4. This app is useful to improve lifestyles	1	2	2	4	4
5. This app helps to better know and understand the importance of healthy lifestyles	1	0	2	7	3
6. I thought this app will be useful if I continue using it	1	1	3	5	3
